# Pharmacist-led guideline-directed medical therapy in heart failure: impact analysis in primary care

**DOI:** 10.1136/bmjoq-2025-003401

**Published:** 2025-09-01

**Authors:** Markus Schichtel, Stephen Barclay, Helena Papworth, Leila Mills, Ben Bowers

**Affiliations:** 1 Department of Public Health and Primary Care, University of Cambridge, Cambridge, England, UK; 2 NHS Cambridgeshire and Peterborough STP, Cambridge, England, UK

**Keywords:** Community Pharmacy Services, Continuous quality improvement, Evaluation methodology, Patient-centred care

## Abstract

Optimal guideline-directed medical therapy (GDMT) can reduce mortality, unplanned hospital admissions and improve quality of life for patients suffering from heart failure (HF). However, GDMT remains underused in primary care. Only a minority of patients on HF registers receive optimal GDMT in the UK. This suboptimal care is compounded by a mounting lack of GP capacity and the growing burden of HF.

A multisite, quantitative impact analysis was undertaken to evaluate the optimisation of HF patients by a novel pharmacist-led GDMT model in UK primary care.

We identified low-risk HF patients suitable for pharmacists’ input, including a community validated risk stratification tool—the HF Event STrengthening Score. The primary outcome was to compare the proportion of patients on optimal HF GDMT at 6 months and 2 years with baseline. Secondary outcomes were direct personnel healthcare costs and GP workload. A subgroup analysis was modelled to estimate effect on mortality, hospitalisation and quality of life.

A total of 237 patients were included. Pharmacist-led GDMT contributed to the increase of optimal GDMT from 17.7% at baseline to 76.5% at 6 months and 94.5% at 2 years follow-up. The novel approach reduced GPs’ HF GDMT workload by 36.6% at 6 months and 42.1% at 2 years and healthcare costs by 18.4% at 6 months and 20.3% at 2 years. Patients with combined angiotensin receptor neprilysin inhibitor/sodium glucose co-transporter 2 inhibitor treatment indicated a reduction of 20.8% in cardiovascular mortality, a reduction of 34.8% in hospitalisations and a 5.31 Kansas City Cardiomyopathy Questionnaire Score for improved quality of life at 2 years.

For low-risk HF patients, pharmacist-led optimisation achieved significantly higher GDMT rates, reduced personnel healthcare costs, reduced GPs’ workload, contributed to reduced cardiovascular mortality, reduced hospitalisations and improved quality of life. In the context of current workload pressures, this approach should be considered for widespread implementation in general practice.

**WHAT IS ALREADY KNOWN ON THIS TOPIC**

- Optimal guideline-directed medical therapy (GDMT) can reduce mortality, unplanned hospital admissions and improve quality of life for patients suffering from heart failure (HF). However, GDMT remains underused in primary care. We evaluated a novel pharmacist-led GDMT model to improve the optimisation of HF patients in UK primary care.

**WHAT THIS STUDY ADDS**

- Pharmacist-led GDMT contributed to the increase of optimal GDMT from 17.7% at baseline to 76.5% at 6 months and 94.5% at the 2-year follow-up. The novel approach reduced GPs’ HF GDMT workload by 36.6% at 6 months and 42.1% at 2 years and healthcare costs by 18.4% at 6 months and 20.3% at 2 years. Patients whose treatment included angiotensin receptor neprilysin inhibitor and sodium glucose co-transporter 2 inhibitor indicated a 20.8% reduction in cardiovascular mortality, a 34.8% reduction in hospitalisations and a 5.31 Kansas City Cardiomyopathy Questionnaire Score for improved quality of life at 2 years.

**HOW THIS STUDY MIGHT AFFECT RESEARCH, PRACTICE OR POLICY**

- Pharmacist-led GDMT *in primary care* has the potential to significantly improve optimal GDMT in HF over a prolonged period, reduce personnel costs, reduce cardiovascular mortality, hospitalisations and improve quality of life. Involving pharmacists in HF GDMT should be considered more widely in primary care.

## Introduction and background

 The burden of heart failure (HF) in the UK is increasing and is now similar to the four most common cancers combined.^1 2^ HF accounts for 2% of the total National Health Service budget, with 70% of these costs due to hospitalisations.^3 4^ Optimal guideline-directed medical therapy (GDMT), a standardised treatment approach for managing patients with HF,^5^ is estimated to reduce unplanned hospital admissions, lower mortality by >70% and improve quality of life.^6 7^ The absence of GDMT can increase the risk of a person dying from HF with reduced ejection fraction (HFrEF) by 29%.^8 9^

In the UK, the HF patient care pathway generally begins with a referral from primary care to a hospital for suspected HF based on clinical symptoms, ECG findings and elevated levels of N-terminal pro-brain natriuretic peptide (NTproBNP).^10^,^12^ If the diagnosis is confirmed, hospital treatment stabilises patients and discharges them back into the community with a treatment plan including GDMT.^2^ However, many HF patients are not yet on optimal therapy at discharge due to several factors including patient drug tolerability, blood pressure or renal function concerns.^12^ General Practitioners (GPs) and other primary healthcare professionals are expected to continue optimising GDMT to target doses as per National Institute for Health and Care Excellence (NICE) or European Society of Cardiology guidelines.^10 12 13^

Hence, primary care has a central role in preventing hospitalisations by providing optimal care for HF patients including GDMT.^14 15^ But GDMT remains underused in general practice.^16^ An analysis by NICE demonstrated that nationally, only the minority of patients on HF registers in primary care are on optimal therapy.^10^ This suboptimal management is compounded by the growing burden of HF and the mounting lack of GP capacity to optimise HF care.^17^,^20^ In 2021/2022, there was an estimated shortage of around 4200 full-time equivalent GPs in England and 200 000 new cases of HF annually.^17 21 22^

Innovative solutions are necessary to address the GP capacity shortage in delivering GDMT and improve patient outcomes.^23^ While the contribution of pharmacists in managing HF GDMT in *secondary* care has long been supported, there is a paucity of data on the impact of pharmacist-led GDMT in *primary* care.^24^,^26^ Furthermore, there is little evidence on how primary care pharmacists could *safely* contribute to HF GDMT. Our aim was therefore to evaluate the optimisation of HF patients by using a novel pharmacist-led GDMT model in primary care.

## Method

### Design, setting and participants

This evaluation involved a retrospective analysis of data at Nene Valley Hodgson Medical Practices (NVH), involving 237 HF patients, covering the period from September 2022 to August 2024. NVH has a practice population of 19 500 patients and is located on two sites in Peterborough, East Anglia, United Kingdom. Patients 18 years or older were included if they had a diagnosis of HF as of 1 September 2022. We (LM and MS) conducted searches using the SystmOne practice database which identifies patients with an active HF diagnosis in their problem list. HF included patients suffering from HFrEF and HF with preserved ejection fraction (HFpEF). HFrEF is a subgroup of HF patients with their left ventricular ejection fraction (LVEF) below 50%.^27^ HFpEF is a class of patients with a normal or near-normal LVEF but elevated natriuretic peptide levels (NTproBNP) and clinical signs of HF such as shortness of breath, oedema or orthopnoea.^28^

### Data extraction, parameters and analysis

Data were extracted by clinical members of the practice team (HP, LM and MS) using structured query language, a standard process for accessing and manipulating relational databases on 01 September 2022, 01 March 2023 and 31 August 2024. Patient characteristics gathered included age, race, gender, New York Heart Association (NYHA) class, LVEF, status of GDMT, up-titration and initiation of beta blockers (BB), ACE inhibitors (ACEI), angiotensin receptor blockers (ARB) and mineralocorticoid antagonist (MRA), angiotensin receptor neprilysin inhibitors (ARNIs), sodium glucose co-transporter 2 inhibitors (SGLT2i), loop diuretics, digoxin, HF Event STrengthening (HFESTOS) score, NTproBNP level and estimated glomerular filtration rate.

Event rates for cardiovascular death, all-cause mortality and HF hospitalisation were approximated from PARADIGM-HF (ARNI vs ACEI)^29^ and DAPA-HF (dapagliflozin vs placebo)^30^ for SLGT2is and ARNI-eligible patients at NVH comparing treated and untreated cohorts over 2 years. HRs were multiplied to estimate combined effects.^31^ Quality of life was assessed via Kansas City Cardiomyopathy Questionnaire (KCCQ)^32^ (see online supplemental appendix 1 for a detailed statistical analysis).

This project was reviewed and deemed exempt from research ethics committee approval by the Research Governance Committee of the Cambridgeshire and Peterborough Integrated Care System. It was determined by the committee that this project was a service evaluation and conducted in accordance with the ethical principles of the Helsinki’s Declaration.^33^ Patient consent was waived as no patient identifiers were collected and the intervention was within the clinical scope of practice for pharmacists and clinicians at NVH. We followed the guidance provided by the authors of the HFESTOS Score which contributed to the identification of low-risk HF patients suitable for pharmacist-led GDMT.^34^

### HFESTOS score

The HFESTOS score was the first externally validated scale to be applied in patients with HF in a *community* setting to risk stratify patients into low, medium and high risk of HF decompensation and included both patients suffering from HFpEF and HFrEF.^34^ The scale is based on variables readily collected in primary care: sex, previous hospitalisation, presence of pulmonary crackles, paroxysmal nocturnal dyspnoea, orthopnoea, presence of NYHA scale functional levels III or IV,^35^ worsening of the NYHA score, heart rate and pulse oximetry. Low-risk HF patients were considered suitable for pharmacist-led GDMT. Patients at medium risk were more appropriate for nurse-led GDMT and patients at high risk were reviewed by GPs.

### Delivery of care

Prior to this project, patients had no dedicated pharmacist or nurse input and had received HF medication up-titrations inconsistently, predominantly from GPs. All pharmacists’ appointments were undertaken as planned telephone appointments. GP and nurse appointments were either conducted by telephone or face-to-face consultations at NVH with access to blood pressure monitors, pulse oximeter, weight scales and ECG. Pharmacists and clinicians could also enter home telehealth consultations which allowed for a community nurse to assist with house-bound patients to measure data such as weight, blood pressure or pulse. In most cases, this allowed for remote and efficient appointments without the need to see patients face-to-face.

### Pharmacists’ competency and responsibilities

The pharmacist-led GDMT was staffed by three practice pharmacists (SH, CB and RK) who underwent additional training including a 4-hour training session on HF GDMT, thresholds for systolic and diastolic blood pressure and pulse rate, identifying signs of decompensation in HF, limits for renal function and pulse oximetry. Their pharmacological responsibilities focused only on the up-titration but not on the initiation of the four pillars of HF treatment: BB, ACE, ARB and MRAs. ARNIs, SGLT2is or loop diuretics were initiated and up-titrated predominantly by a GP (MS) or nurse (JNW and RM). Pharmacists were able to order necessary laboratory tests and had access to senior clinicians for any management queries and immediate patient-related needs. Additionally, pharmacists monitored and interpreted blood results, surveyed blood pressure, pulse and drug side effects and provided a limited amount of patient education.

### Follow-up frequency

The frequency of up-titration appointments depended on the medication-specific changes. Exceptions were those patients who developed drug sensitivities, declined to take medication or for whom no further pharmacological option existed within the limits of national guidelines.

### Outcomes

The primary outcomes were the proportion of patients who achieved optimal GDMT at 6 months and 2 years including ACEI/ARB/ARNI, beta-blocker, MRAs, SGLT2is and digoxin.

Optimal GDMT was defined as achieving the target doses of NICE-recommended HF medications or reaching a maximum dose^36^ beyond which additional dose titrations could not be safely achieved due to adverse effects such as low blood pressure (<100/60 mm Hg), low heart rate (< 50 beats per minute) or signs and symptoms of hypotension.

Secondary outcomes consisted of the difference in direct personnel healthcare costs comparing the standard model with the new service approach at 6 months and 2 years. A cost analysis (HP and MS) was conducted to assess the difference between pharmacist-led GDMT and standard practice. Cost of pharmacist-led GDMT included direct personnel cost, additional follow-up appointments and supervisory requirements. We subtracted the cost of pharmacist-led GDMT from the estimated cost of GP GDMT. Cost of the pharmacist intervention was calculated as direct personnel cost as total number of appointments time (20 min) per appointment×£12.50. Additional follow-up appointments were added as part of pharmacists’ direct personnel costs. Any further overheads such as patient education materials, space or electricity were assumed to be minimal and therefore were not included in the analysis. Net benefit cost reductions at 6 months and 2 years were calculated as total cost savings minus total costs. Categorical data were predominantly represented as proportions and averages.

To model the combined effect of ARNIs and SGLT2is for eligible patients, we assumed additive benefits on the log-hazard scale, which translates to multiplying HRs from the PARADIGM-HF and DAPA-HF trials, a standard approach for combining independent treatment effects when no significant interaction is reported.^31^

## Results

A total of 237 patients were identified at the 2-year follow-up: a 50% increase from 158 HF patients identified at baseline. The median age at baseline was 75 years (age range 39–96 years). Most patients were white, male, NYHA Class II and a LVEF between 40–49% (see online supplemental appendix for details).

The practice HF prevalence was 1.21%. The most common comorbidities were chronic kidney disease (CKD), hypertension, type 2 diabetes mellitus, atrial fibrillation and ischaemic heart disease, followed by chronic obstructive pulmonary disease, myocardial infarction, stroke and coronary artery bypass graft (see online supplemental appendix 2).

**Figure 1:**
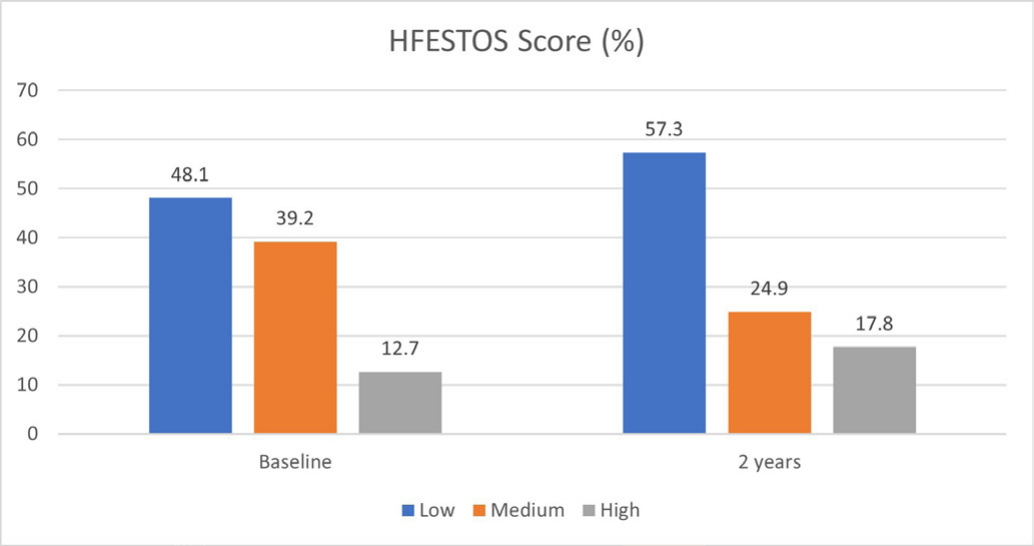
HF Event STrenthening Score.

At baseline, 48.1% of patients had a low risk HFESTOS score, 39.2% were classified as medium risk and 12.7% as high risk. At the 2-year follow-up, most HF patients were rated as low risk (57.3%) (figure 1).

### Primary outcome

**Figure 2:**
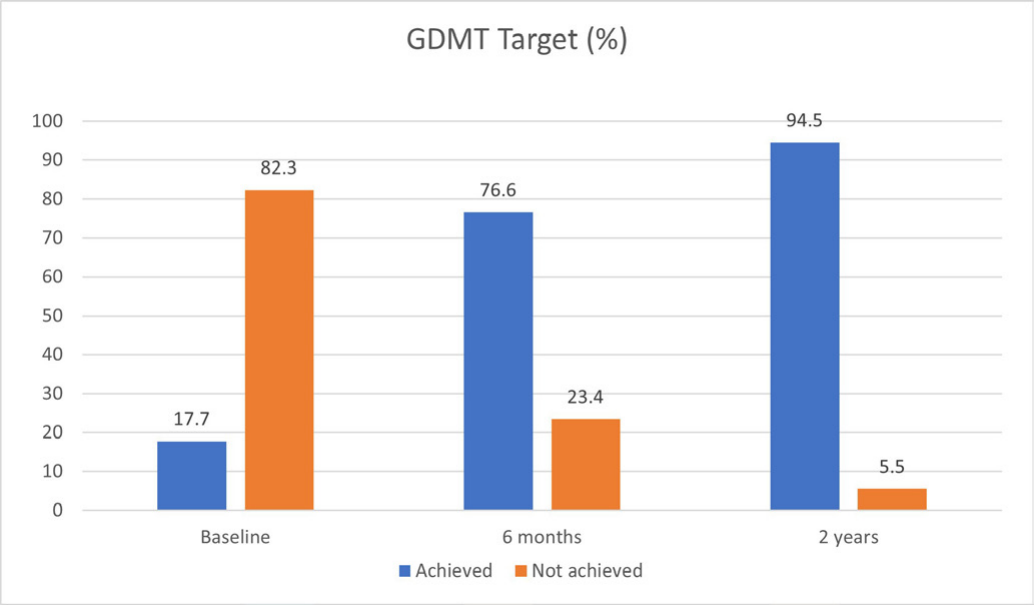
Guideline-directed medical therapy status.

The proportion of patients on optimal GDMT at baseline was 17.7% (figure 2). This rose to 76.6% at 6 months and 94.5% at 2 years.

**Figure 3:**
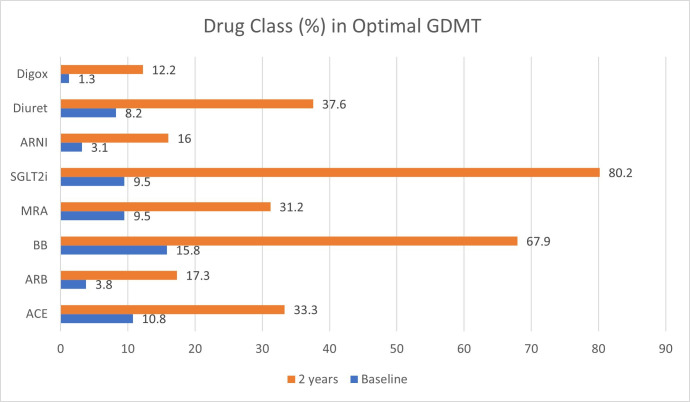
Drug Class in Optimal GDMT. ARB, angiotensin receptor blockers; ARNI, angiotensin receptor neprilysin inhibitor; BB, beta blockers; GDMT, guideline-directed medical therapy; SGLT2i, sodium glucose co-transporter 2 inhibitor MRA, mineralocorticoid antagonist.

Drug class proportions in optimal GDMT increased significantly at 2-year follow-up compared with baseline: SGLT2is (9.5% vs 80.2%), ACE (10.8% vs 33.3%), ARB (3.8% vs 17.3%), ARNIs (3.1% vs 16%), BBs (15.8% vs 67.9%) and MRAs (9.5% vs 31.2%) (figure 3).

The process of up-titration was not yet completed in 15 patients (6.32%) at 2 years mainly because those were newly added to the HF service. Other common reasons for not reaching target GDMT were adverse drug effects including hypotension, diarrhoea, dysuria, bradycardia or hyperkalaemia.

### Secondary outcomes

Pharmacists covered the greatest proportions of HF GDMT at 6 months (36.6%) and 2 years (42.1%) compared with nurses (32.9% and 30.3%) and GPs (29.7% and 35.0%), respectively.

A cohort of 202 HF patients eligible for dapagliflozin, including 40 patients eligible for ARNIs, was identified. At baseline, only 9.5% of eligible patients were on dapagliflozin and 3.15% of eligible patients received ARNI. At 2 years, compared with the untreated baseline, the treated cohort had five fewer cardiovascular deaths (19 vs 24; 20.8% reduction), six fewer all-cause deaths (23 vs 29; 20.7% reduction) and eight fewer hospitalisations (15 vs 23; 34.8% reduction). KCCQ scores improved by 3.87 points more in the treated cohort (5.31 vs 1.44), with 49 more patients achieving ≥5-point improvement (106 vs 57).

### Cost analysis

The average appointment total per patient was 3.25 appointments at 6 months (pharmacist 1.19, nurse 1.08, GP 0.98 appointments) and 4.17 at 2-year follow-up (pharmacist 1.76, nurse 1.17, GP 1.24 appointments) (see online supplemental appendix for details).

Pharmacists undertook a total of 192 telephone appointments at 6 months and 408 appointments at 2 years lasting on average 20 min each. Pharmacist required a total of four GP supervisory sessions at 6 months. Supervisory requirements at 2 years were minimal and not included in the analysis. Their contribution resulted in a total of 48 hours (12 sessions) of GP time saved at 6 months and 136 hours (34 sessions) of GP time saved at 2 years. Based on the number of individual contacts per patient including additional costs, this approach reduced direct personnel costs by 18.4% at 6 months and 20.3% at 2 years.

## Discussion

### Summary

The project demonstrated that a considerably higher proportion of HF patients achieved optimal GDMT with pharmacist-led input from baseline (17.7%) over a sustained period of 2 years (94.5%). The HFESTOS score was useful in identifying low risk HF patients suitable for pharmacist-led GDMT. Pharmacist involvement reduced direct personnel costs by 18.4% at 6 months and 20.3% at 2 years. These findings correspond with previously published studies on the impact of pharmacist involvement in secondary care showing a significant association between achieving optimal GDMT and a reduction in unplanned hospital admissions, emergency department visits and healthcare cost.^37^,^40^ No such data are available from primary care to date.

The estimated 20.8% reduction in cardiovascular mortality aligns with trial-reported benefits (20% for ARNI, 18% for dapagliflozin)^29 30^ and suggests additive effects, consistent with DAPA-HF subgroup findings. The 3.87-point KCCQ improvement, while statistically significant, may not universally reach clinical significance (≥5 points), but reduced hospitalisations of 34.8% enhance patient well-being. These findings highlight the potential synergistic benefits of ARNIs and SGLT2is in HF management, warranting further real-world validation.

For patients, the combined therapy offers a lower risk of death, fewer hospital stays and a higher likelihood of feeling better (less dyspnoea, fatigue and functional limitation). These outcomes align with patient priorities: living longer, staying out of the hospital and maintaining functional independence.

### Strengths and limitations

Pharmacist-led GDMT provided a structured approach to medication management, ensuring continuity of care and reducing the risk of medication errors or gaps.^41^ Since pharmacists were working according to clearly defined up-titration protocols, patients received appropriate medications at appropriate doses and at recommended time intervals, reducing the risk of adverse events and improving treatment outcomes.^42^ After hospital discharge, HF patients also had earlier access to pharmacist-led GDMT at the practice. This approach has been shown to help reduce the risk of readmissions and lead to significant cost savings in a previous study.^25^ As a result of pharmacists managing over a third of the GDMT workload, GPs had more capacity to focus on more complex HF cases, including a greater consideration for initiating ARNIs and SGLT2is, which led to more frequent prescriptions. Both medication classes were previously underutilised at the practice but have a robust evidence base to lower hospitalisations and mortality.^29 43 44^

The HFESTOS score was based on variables readily collected in primary care.^34^ Its risk classification proved to be useful in identifying low risk HF patients for pharmacist-led GDMT. While the HFESTOS score does not directly address cost benefit calculations, its risk-stratification capabilities would seem to have potential to inform human resource allocation, leading to cost reductions. Further research is needed to fully explore the relationship between HFESTOS score, human resource allocation and corresponding healthcare costs.

This evaluation had a modest sample size, with a non-randomised study design and did not have a comparative group. However, we were able to demonstrate the sustained impact of pharmacists on GDMT in primary care over a 2-year period; a much longer duration than published studies from secondary care.^25 37^ The large proportion of white British male patients limits the generalisability of findings. The primary outcome was optimal guideline concordant GDMT prescribing. This is a proxy marker associated with a reduction in unplanned hospital admissions, reduced mortality and better quality of life^6^,^9^. These clinical outcomes were part of the modelling analysis.

The prevalence of HF was at the higher end of the national average of 0.8–1.2% in general practice^11 21 45^ due to high socioeconomic deprivation and associated greater burden of comorbidities.^46 47^ The practice patients identified with HF at NVH increased significantly during the 2-year period, which may be related to a number of factors including:

- Earlier diagnosis of HF due to upskilled primary care staff involving pharmacists and nurses leading to improved diagnostic capabilities and earlier diagnostic tests such as NTproBNP and echocardiograms.^48^
- Effectively capturing the HF practice population by combining regular searches on SystmOne with an accurate and detailed coding practice^49^ to specify the HF diagnosis in a HF database.
- Genuine growth in the prevalence of HF in line with regional and national trends, related to an ageing population and a rising incidence of cardiovascular comorbidities such as hypertension, type 2 diabetes, obesity and CKD.^50^

A potential weakness of the pharmacist-led HF GDMT included their limited scope of practice. At the start of the new service, none were confident in initiating new classes of HF drugs and only addressed up-titration of existing medication. Additionally, there was some variability in pharmacists’ expertise in HF-specific GDMT. We identified and addressed these issues with case-based training tailored to the specific learning needs of individual clinicians. One pharmacist needed closer physician supervision and approval as they were not yet a non-medical prescriber. We coordinated this pharmacist’s GDMT clinic with that of a GP to provide more immediate support and avoid any delays or variability in the implementation of GDMT.

## Conclusions and implications for research and practice

Pharmacist-led GDMT in primary care has the potential to significantly improve optimal GDMT in HF over a prolonged period, reduce personnel costs, improve cardiovascular mortality, hospitalisations and quality of life. The HFESTOS score appears useful in identifying low-risk HF patients suitable for pharmacists’ input. Involving pharmacists in HF GDMT should be considered more widely in primary care. Future randomised controlled trials should directly evaluate the association between HFESTOS and healthcare resource allocations and ARNI+SGLT2 i combinations in primary care to confirm these findings.

## Supplementary material

10.1136/bmjoq-2025-003401 online supplemental file 1

10.1136/bmjoq-2025-003401 online supplemental file 2

## Data Availability

All data relevant to the study are included in the article or uploaded as supplementary information.

## References

[1] Anwar MS, Japp AG, Mills NL (2019). Heart failure and healthcare informatics. PLoS Med.

[2] Conrad N, Molenberghs G, Verbeke G (2024). Trends in cardiovascular disease incidence among 22 million people in the UK over 20 years: population based study. BMJ.

[3] Stewart S, Jenkins A, Buchan S (2002). The current cost of heart failure to the National Health Service in the UK. Eur J Heart Fail.

[4] Lesyuk W, Kriza C, Kolominsky-Rabas P (2018). Cost-of-illness studies in heart failure: a systematic review 2004-2016. BMC Cardiovasc Disord.

[5] Patel J, Rassekh N, Fonarow GC (2023). Guideline-Directed Medical Therapy for the Treatment of Heart Failure with Reduced Ejection Fraction. Drugs (Abingdon Engl).

[6] Tang AB, Ziaeian B, Butler J (2024). Global Impact of Optimal Implementation of Guideline-Directed Medical Therapy in Heart Failure. JAMA Cardiol.

[7] Roth GA, Poole JE, Zaha R (2016). Use of Guideline-Directed Medications for Heart Failure Before Cardioverter-Defibrillator Implantation. J Am Coll Cardiol.

[8] McCullough PA, Mehta HS, Barker CM (2021). Mortality and guideline-directed medical therapy in real-world heart failure patients with reduced ejection fraction. Clin Cardiol.

[9] Naegele M, Flammer AJ, Enseleit F (2016). Medical therapy of heart failure with reduced ejection fraction: current evidence and new developments. Swiss Med Wkly.

[10] NICE (2018). Chronic heart failure in adults: diagnosis and management. https://www.nice.org.uk/guidance/ng106.

[11] Taylor CJ, Rutten FH, Brouwer JR (2017). Practical guidance on heart failure diagnosis and management in primary care: recent EPCCS recommendations. Br J Gen Pract.

[12] Bottle A, Kim D, Aylin PP (2018). Real-world presentation with heart failure in primary care: do patients selected to follow diagnostic and management guidelines have better outcomes?. Open Heart.

[13] McDonagh TA, Metra M, Adamo M (2021). 2021 ESC Guidelines for the diagnosis and treatment of acute and chronic heart failure. Eur Heart J.

[14] Lynch KA, Ganz DA, Saliba D (2022). Improving heart failure care and guideline-directed medical therapy through proactive remote patient monitoring-home telehealth and pharmacy integration. *BMJ Open Qual*.

[15] Mold J (2017). Goal-Directed Health Care: Redefining Health and Health Care in the Era of Value-Based Care. Cureus.

[16] Newman E, Kamanu C, Gibson G (2024). How to Optimize Goal-Directed Medical Therapy (GDMT) in Patients with Heart Failure. Curr Cardiol Rep.

[17] NHS (2024). General practice workforce: national health service digital. https://digital.nhs.uk/data-and-information/publications/statistical/general-and-personal-medical-services.

[18] THF (2023). General practice data dashboard 07/03/2023: the health foundation. https://www.health.org.uk/reports-and-analysis/analysis/general-practice-data-dashboard.

[19] Heidenreich P (2024). Heart failure management guidelines: New recommendations and implementation. J Cardiol.

[20] Norhammar A, Bodegard J, Vanderheyden M (2023). Prevalence, outcomes and costs of a contemporary, multinational population with heart failure. Heart.

[21] Cowie MR (2017). The heart failure epidemic: a UK perspective. Echo Res Pract.

[22] BHF (2021). Heart failure - a blueprint for change: British heart foundation. https://www.bhf.org.uk/what-we-do/policy-and-public-affairs/transforming-healthcare/heart-failure-report.

[23] RCGP (2019). Fit for the future London: royal college of general practitioners. https://www.rcgp.org.uk/getmedia/ff0f6ea4-bce1-4d4e-befc-d8337db06d0e/RCGP-fit-for-the-future-report-may-2019.pdf.

[24] Zheng J, Mednick T, Heidenreich PA (2023). Pharmacist- and Nurse-Led Medical Optimization in Heart Failure: A Systematic Review and Meta-Analysis. J Card Fail.

[25] Patil T, Ali S, Kaur A (2022). Impact of Pharmacist-Led Heart Failure Clinic on Optimization of Guideline-Directed Medical Therapy (PHARM-HF). J Cardiovasc Transl Res.

[26] Shah SP, Dixit NM, Mendoza K (2022). Integration of clinical pharmacists into a heart failure clinic within a safety-net hospital. J Am Pharm Assoc (2003).

[27] Nadar SK, Tariq O (2018). What is Heart Failure with Mid-range Ejection Fraction? A New Subgroup of Patients with Heart Failure. *Card Fail Rev*.

[28] Harper AR, Patel HC, Lyon AR (2018). Heart failure with preserved ejection fraction. Clin Med (Lond).

[29] McMurray JJV, Packer M, Desai AS (2014). Angiotensin-neprilysin inhibition versus enalapril in heart failure. N Engl J Med.

[30] McMurray JJV, Solomon SD, Inzucchi SE (2019). Dapagliflozin in Patients with Heart Failure and Reduced Ejection Fraction. *N Engl J Med*.

[31] Moodie PF, Saville BR, Koch GG (2011). Estimating Covariate-Adjusted Log Hazard Ratios for Multiple Time Intervals in Clinical Trials Using Nonparametric Randomization Based ANCOVA. Stat Biopharm Res.

[32] Nikolaou M, Parissis J, Farmakis D (2006). Clinical and prognostic implications of Kansas City Cardiomyopathy Questionnaire in patients with chronic heart failure. Eur Heart J.

[33] World Medical A (2013). World Medical Association Declaration of Helsinki: ethical principles for medical research involving human subjects. JAMA.

[34] Verdu-Rotellar J-M, Abellana R, Vaillant-Roussel H (2022). Risk stratification in heart failure decompensation in the community: HEFESTOS score. ESC Heart Fail.

[35] Cosiano MF, Tobin R, Mentz RJ (2021). Physical Functioning in Heart Failure With Preserved Ejection Fraction. J Card Fail.

[36] Malgie J, Clephas PRD, Brunner-La Rocca H-P (2023). Guideline-directed medical therapy for HFrEF: sequencing strategies and barriers for life-saving drug therapy. Heart Fail Rev.

[37] Polsinelli VB, Sun J-L, Greene SJ (2024). Hospital Heart Failure Medical Therapy Score and Associated Clinical Outcomes and Costs. JAMA Cardiol.

[38] Dixit NM, Shah S, Ziaeian B (2021). Optimizing Guideline-directed Medical Therapies for Heart Failure with Reduced Ejection Fraction During Hospitalization. *US Cardiol*.

[39] Balakumaran K, Patil A, Marsh S (2019). Evaluation of a guideline directed medical therapy titration program in patients with heart failure with reduced ejection fraction. Int J Cardiol Heart Vasc.

[40] Margolin E, Huynh T, Brann A (2024). Determinants of Guideline-Directed Medical Therapy Implementation During Heart Failure Hospitalization. *JACC Adv*.

[41] Bethishou L, Herzik K, Fang N (2020). The impact of the pharmacist on continuity of care during transitions of care: A systematic review. J Am Pharm Assoc (2003).

[42] Zhang Z, Wang C, Tu T (2024). Advancing Guideline-Directed Medical Therapy in Heart Failure: Overcoming Challenges and Maximizing Benefits. Am J Cardiovasc Drugs.

[43] Tai C, Gan T, Zou L (2017). Effect of angiotensin-converting enzyme inhibitors and angiotensin II receptor blockers on cardiovascular events in patients with heart failure: a meta-analysis of randomized controlled trials. BMC Cardiovasc Disord.

[44] Ali AE, Mazroua MS, ElSaban M (2023). Effect of Dapagliflozin in Patients with Heart Failure: A Systematic Review and Meta-Analysis. Glob Heart.

[45] Bellanca L, Linden S, Farmer R (2023). Incidence and prevalence of heart failure in England: a descriptive analysis of linked primary and secondary care data - the PULSE study. BMC Cardiovasc Disord.

[46] ONS (2021). Local indicators for Peterborough: office of national statistics. https://explore-local-statistics.beta.ons.gov.uk/areas/E06000031-peterborough/indicators.

[47] Hawkins NM, Jhund PS, McMurray JJV (2012). Heart failure and socioeconomic status: accumulating evidence of inequality. Eur J Heart Fail.

[48] Taylor CJ (2023). Earlier heart failure diagnosis in primary care. Br J Gen Pract.

[49] Walkey AJ, Shieh M-S, Pekow P (2019). Changing Heart Failure Coding Practices and Hospital Risk-Standardized Mortality Rates. J Card Fail.

[50] Emmons-Bell S, Johnson C, Roth G (2022). Prevalence, incidence and survival of heart failure: a systematic review. Heart.

