# Supplementary material for: Pharmacist-led guideline-directed medical therapy in heart failure: impact analysis in primary care
Source: BMJ Open Qual. 2025 Sep 1;14(3):e003401. doi: 10.1136/bmjoq-2025-003401 (PMC12406906; doi:10.1136/bmjoq-2025-003401)
Supplement: online supplemental file 1 [file bmjoq-14-3-s001.docx]

## **Suppl. Appendix 1.**

To estimate the combined effect of ARNIs and SGLT2is (Dapagliflozin) on mortality, hospitalization, and quality of life for the cohort of 202 heart failure patients (40 ARNI-eligible), I employed a series of statistical and analytical methods based on the data from the PARADIGM-HF and DAPA-HF trials. Below, I outline the specific statistical methods used to calculate the outcomes.

### 1. **Cohort Proportions and Event Rate Estimation**

- **Method**: **Proportion Calculation and Rounding**
  - Baseline and 2-year treatment proportions (e.g., 9.5% on dapagliflozin at baseline, 94.5% at 2 years; 3.15% on ARNIs at baseline, 94.5% at 2 years).
  - I calculated the number of patients in each treatment group by multiplying the cohort size (202 for dapagliflozin, 40 for ARNIs) by these proportions (e.g., 0.095 × 202 = 19.19, rounded to 19 patients).
  - Rounding was applied to convert fractional patients to whole numbers for practical interpretation, as fractional events or patients are not meaningful in a clinical context.

### 2. **Event Rate Extrapolation for Two-Year Outcomes**

- **Method**: **Approximation from Kaplan-Meier Estimates**
  - The PARADIGM-HF trial (median follow-up 27 months) and DAPA-HF trial (median follow-up 18.2 months) did not directly report 2-year (24-month) event rates for all outcomes.
  - I approximated 2-year event rates using published Kaplan-Meier survival curves and supplementary data:
    - **PARADIGM-HF**: Cardiovascular death (~11.5% ARNI, ~14.5% ACEI), all-cause mortality (~14.5% ARNI, ~17.0% ACEI), hospitalization for heart failure (~10.5% ARNI, ~13.0% ACEI).
    - **DAPA-HF**: Cardiovascular death (~10.5% dapagliflozin, ~12.5% placebo), all-cause mortality (~12.5% dapagliflozin, ~15.0% placebo), hospitalization for heart failure (~8.5% dapagliflozin, ~12.5% placebo).
  - These rates were derived by visually inspecting Kaplan-Meier curves (e.g., Figure 2 in McMurray et al., 2019 for DAPA-HF) and aligning with reported hazard ratios (HRs) and event rates at earlier time points (e.g., 18.2 months for DAPA-HF).
  - For DAPA-HF, I assumed a constant hazard rate beyond 18.2 months to extrapolate to 24 months, supported by the stable HRs observed in the trial.

### 3. **Combining Treatment Effects (ARNIs + Dapagliflozin)**

- **Method**: **Multiplicative Hazard Ratio Model**
  - To estimate the combined effect of ARNIs and dapagliflozin for the 40 ARNI-eligible patients, I assumed additive benefits on the log-hazard scale, which translates to multiplying hazard ratios (HRs) from the two trials. This is a standard approach for combining independent treatment effects when no significant interaction is reported.
  - HRs were obtained from trial results:
    - **PARADIGM-HF**: Cardiovascular death HR = 0.80, all-cause mortality HR = 0.84, hospitalization HR = 0.79.
    - **DAPA-HF**: Cardiovascular death HR = 0.82, all-cause mortality HR = 0.83, hospitalization HR = 0.70.
  - Combined HRs for the ARNI-eligible subgroup:
    - Cardiovascular death: 0.80 × 0.82 = 0.656.
    - All-cause mortality: 0.84 × 0.83 = 0.6972.
    - Hospitalization: 0.79 × 0.70 = 0.553.
  - The combined HR was applied to the baseline event rate (from the untreated arm, typically DAPA-HF placebo or PARADIGM-HF ACEI) to estimate the treated event rate:
    - Example: Baseline cardiovascular death rate (PARADIGM-HF ACEI, 14.5%) × 0.656 = 9.512% for ARNI + dapagliflozin.

### 4. **Event Number Calculation**

- **Method**: **Expected Value Calculation**
  - For each outcome (mortality, hospitalization), I calculated the expected number of events by multiplying the event rate by the number of patients in each subgroup:
    - Example (ARNI-eligible, untreated): Cardiovascular death rate (14.5%) × 39 patients = 0.145 × 39 = 5.655 ≈ 6 patients.
    - Example (ARNI-eligible, treated): Combined rate (9.512%) × 38 patients = 0.09512 × 38 = 3.61456 ≈ 4 patients.
  - Rounding was applied to ensure whole numbers for events, reflecting clinical interpretability.

### 5. **Quality of Life (KCCQ) Estimation**

- **Method**: **Additive Mean Change and Proportion Analysis**
  - **Mean KCCQ Score Change**:
    - For PARADIGM-HF, ARNI resulted in a ~1.0–1.5-point less decline compared to ACEI (~2.5-point decline).
    - For DAPA-HF, dapagliflozin resulted in a ~5.0–5.5-point improvement compared to ~2.5-point improvement for placebo.
    - For the ARNI-eligible subgroup, I combined effects additively: PARADIGM-HF (~1.0-point decline) + DAPA-HF (~5.5-point improvement) = ~4.5-point net improvement.
    - For the non-ARNI-eligible subgroup, I used DAPA-HF alone (~5.5-point improvement).
    - Weighted average for the combined cohort: (38/191 × 4.5) + (153/191 × 5.5) ≈ 5.31-point improvement (treated) vs. (39/183 × –2.5) + (144/183 × 2.5) ≈ 1.44-point improvement (untreated).
  - **Clinically Significant Changes**:
    - Proportions with ≥5-point improvement or decline were taken from trial data:
      - PARADIGM-HF: Clinically significant decline ~25% (ARNI) vs. ~30% (ACEI).
      - DAPA-HF: Improvement ~58% (dapagliflozin) vs. ~51% (placebo); decline ~25% vs. ~33%.
    - For the ARNI-eligible subgroup, I conservatively estimated combined proportions (e.g., ~20% decline, ~60% improvement) based on overlapping benefits.
    - Expected patients were calculated: e.g., 0.60 × 38 = 22.8 ≈ 23 patients with significant improvement.

### 6. **Subgroup and Combined Cohort Analysis**

- **Method**: **Weighted Aggregation**
  - The cohort was divided into:
    - ARNI-eligible (40 patients): Combined ARNI + dapagliflozin effects.
    - Non-ARNI-eligible (162 patients): Dapagliflozin effects only.
  - Outcomes were calculated separately for each subgroup and then aggregated for the combined cohort:
    - Example (cardiovascular death, treated): (4 × 38/40) + (16 × 153/162) ≈ 19 patients.
    - Untreated cohort was adjusted for baseline proportions (183/202 for dapagliflozin, 39/40 for ARNIs).
  - Weighted averages accounted for subgroup sizes (e.g., 38/191 for ARNI-eligible treated, 153/191 for non-ARNI-eligible treated).

### 7. **Difference Calculation**

- **Method**: **Simple Subtraction**
  - Differences in events (deaths, hospitalizations) and KCCQ outcomes were calculated by subtracting treated cohort estimates from untreated baseline estimates:
    - Example: Cardiovascular deaths (24 untreated – 19 treated = 5 fewer deaths).
    - KCCQ score difference: ~5.31 – 1.44 ≈ 3.87 points.
    - Patients with significant KCCQ changes: e.g., 106 – 57 = 49 more with improvement.
