# Supplementary material for: Pharmacist-led guideline-directed medical therapy in heart failure: impact analysis in primary care
Source: BMJ Open Qual. 2025 Sep 1;14(3):e003401. doi: 10.1136/bmjoq-2025-003401 (PMC12406906; doi:10.1136/bmjoq-2025-003401)
Supplement: online supplemental file 2 [file bmjoq-14-3-s002.docx]

**Suppl. Appendix 2**

1. **Baseline patient characteristics**

| Data parameter | N = 158 |
| --- | --- |
| Age [median (range)]  STDE.P | 75.0 (39-96)  11.66 |
| Sex (male)  (female) | 84 (53.2%)  74 (46.8%) |
| Ethnicity (White)  (Asian)  (Black) | 153 (96.8%)  3 (1.9%)  2 (1.3%) |
| NYHA Class I  II  III  IV  unknown | 12 (7.6%)  75 (47.5%)  62 (39.2%)  5 (3.2%)  4 (2.5%) |
| Left ventricular ejection fraction  >50%  40-49%  30-39%  <30%  unknown | 22 (13.9%)  61 (38.6%)  51 (32.3%)  16 (10.1%)  8 (5.1%) |

1. **Heart failure co-morbidities (%)**


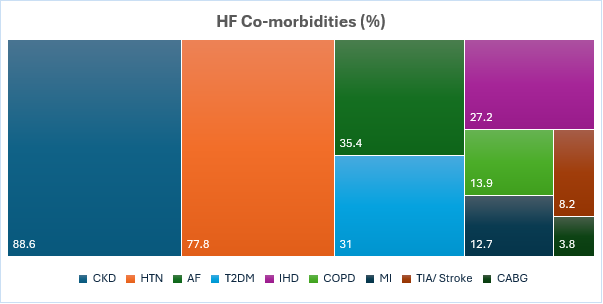


1. **Average number of appointments**
